# Lessons from other diseases: granulomatous inflammation in leishmaniasis

**DOI:** 10.1007/s00281-015-0548-7

**Published:** 2015-12-17

**Authors:** Paul M. Kaye, Lynette Beattie

**Affiliations:** Centre for Immunology and Infection, Department of Biology and Hull York Medical School, University of York, Heslington, York, YO10 5DD UK; QIMR Berghofer Medical Research Institute, 300 Herston Rd, Herston, Queensland Australia 4006

**Keywords:** *Leishmania*, Granuloma, Mouse models, Intravital imaging, Computational models

## Abstract

The *Leishmania* granuloma shares some, though not all, properties with that formed following mycobacterial infection. As a simplified, noncaseating granuloma composed of relatively few and largely mononuclear cell populations, it provides a tractable model system to investigate intra-granuloma cellular dynamics, immune regulation, and antimicrobial resistance. Here, the occurrence of granulomatous pathology across the spectrum of leishmaniasis, in humans and animal reservoir hosts, is first described. However, this review focuses on the process of hepatic granuloma formation as studied in rodent models of visceral leishmaniasis, starting from the initial infection of Kupffer cells to the involution of the granuloma after pathogen clearance. It describes how the application of intravital imaging and the use of computational modeling have changed some of our thoughts on granuloma function, and illustrates how host-directed therapies have been used to manipulate granuloma form and function for therapeutic benefit. Where appropriate, lessons that may be equally applicable across the spectrum of granulomatous diseases are highlighted.

**Electronic supplementary material:**

The online version of this article (doi:10.1007/s00281-015-0548-7) contains supplementary material, which is available to authorized users.

## Leishmaniasis: a complex granulomatous disease

The leishmaniases represent some 15 or so human diseases associated with infection by protozoan parasites of the genus *Leishmania*. Classification of the leishmaniases is not straightforward. Often referred to as a spectrum of diseases, this term implies a continuum in the clinical spectrum associated with a single etiological agent, e.g., as seen for *Mycobacterium leprae* infection [1]. Most forms of leishmaniasis do show a spectrum of responses after infection, from the maintenance of an asymptomatic subclinical infection through to the expression of overt clinical disease with varying degrees of severity. But for the most part, this spectrum exists independently of the well-described clinical boundaries that distinguish cutaneous, metastatic, and visceral manifestations of the disease. Hence, it may be more informative to refer to the leishmaniases as a “collection” of distinct diseases with different etiological agents, each with characteristic and different immune responses and host–pathogen interactions and each having a spectrum of clinical responses [2, 3]. Different species (and subgenera) of *Leishmania* characterize disease in the Old World (Europe, Asia, Africa) and New World (the Americas), reflecting a combination of evolutionary adaptation and geographic dispersal of *Leishmania* parasites and their hosts [4, 5]. Granulomas have been identified following infection with all species of *Leishmania*, often associated with the curative phase of infection.

## Granulomas as hallmarks of protective immunity: an overview

Some of the most comprehensive studies of the *Leishmania* granuloma were conducted by Ridley and Ridley in the early 1980s, who compared different forms and stages of human cutaneous leishmaniasis using a similar approach to that adopted in their pioneering histopathological description of the leprosy spectrum.

They defined many features still relevant today [1]; granulomatous inflammation, mainly showing signs of focal macrophage activation, with or without epitheliod cells, and parasite destruction, in the absence of neutrophil infiltration; and granulomas with evidence of macrophage/monocyte lysis and the presence, albeit transiently, of neutrophils leading to focal necrosis. However, a more contemporary study of CL cases found a surprisingly high number of caseating granulomas (equating to the Ridley group C) [6] It is important to note that the CL lesion has all the hallmarks of a “wound” and as such, the histopathological features of wound healing, including necrotic granulation tissue, fibrinogenesis, and sloughing off of the lesion, occur in parallel with the antimicrobial functions that are associated with granulomatous inflammation. In other words, “lesion” healing may be independent from parasite killing. This notion is supported by the observation that in genetic crosses of *Leishmania major*-susceptible and *Leishmania major-*resistant BALB/c and C57BL/6 mice, genetic loci associated with disease severity did not associate with levels of local immune response, including the extent of granulomatous inflammation [7, 8].

Granulomas with evident multinucleated and epitheliod cells are associated with good in vitro recall responses (measured using the lymphocyte transformation test, LTT) and a requirement for less drug to achieve healing in *Leishmania braziliensis* infection, whereas the presence of necrosis and a polymorph infiltrate was contraindicated [9]. Lymphocyte, plasma cells, and occasional eosinophils also predominate in granulomas in simian models of CL. In *L. braziliensis* infections in the Rhesus monkey (*Macaca mulatta*), chronic granulomas with all the normal human cellular constituents (Langhans giant cells, epitheliod cells, plasma cells, parasite-containing macrophages, lymphocytes) are seen, and at the final stages of healing there is fibroblast infiltration and fibrotic substitution [10, 11].

In contrast to the skin, the characteristic wound healing response is rather minimal in systemic sites, providing the opportunity to examine the antimicrobial functions of granulomas largely independently from this process. However, detailed studies of granulomas in human systemic disease are few and far between, reflecting the difficulties in sampling these sites for histopathological investigation. In humans, the histopathology of active clinical kala-azar most commonly reflects what might be seen as a failure of granulomatous inflammation, being associated with extensive lymphoid tissue remodeling, splenomegaly/lymphadenopathy, and elevated levels of plasma cells [12]. Nevertheless, in some cases, fibrin-ring granulomas (so named for the donut like appearance due to a ring of fibrinoid material) may be evident in the liver [13]. A retrospective analysis of liver biopsies in Iran also suggests that hepatic granulomas may form in some patients with visceral leishmaniasis [14]. Mirroring the absence of granulomas in the spleen of active visceral leishmaniasis (VL) cases, epitheliod granulomas, common in the lymph nodes of CL patients, are also not usually present in Sudanese VL patients who present with lymphadenopathy [15]. Granulomas, usually poorly formed, are also sparse in the bone marrow [16] [17]. In contrast, asymptomatic patients have been reported to have epitheliod granulomas in the liver [18].

In Rhesus monkeys, however, the kinetics of hepatic granuloma formation has been amenable to study. Six weeks post-experimental infection with 2 × 10^7^ amastigotes/kg of body weight of a virulent *Leishmania infantum* strain, poorly differentiated granulomas were observed, largely comprising aggregates of macrophages and lymphocytes. In contrast, by 24 weeks, granuloma organization with the occurrence of Langhans-type multinucleated cells was observed, with evident presence of amastigotes. Although this time course of infection was not extended to determine the rate of involution of granulomas under these infection conditions, animals receiving an experimental vaccine for VL (comprising Ad5-expresing A2 antigen boosted with rA2 + IL-12 + alum) showed granuloma resolution at week 24 along with reduced parasite burden. In the spleens of infected animals, a similar pattern to human disease was noted, including heavily infected macrophages in the subcapsular region and red pulp, disorganized follicular structures, and sinusoidal congestion. Again, vaccinated animals had more normal follicular histology.

In canine VL, both skin [19] and hepatic [20] granuloma formation has been extensively characterized, demonstrating a similar trend to that observed in humans and macaques. Thus, asymptomatic dogs with low parasite burdens had histopathological evidence of robust granuloma formation in the liver, with evidence of recruitment of effector T cells and local macrophage/dendritic cell activation. Granulomas were either not evident or not organized in the liver of symptomatic dogs with high parasite burdens [20]. Of note, whereas tissue response correlated well with parasite load and immune status in the liver, this appeared less evident in the spleen, mirroring the organ-specific dichotomy of responses observed in murine models of VL (see below).

In hamsters, intradermal promastigote inoculation lead to neutrophil influx over the first 48 h followed by a mononuclear cell-rich granuloma that was coincident with clearance of parasites from the skin [21, 22]. Nevertheless, this local response did not stop dissemination and the ultimate development of VL in this model. It was also suggested from these studies that visceral dissemination might need to occur before granuloma formation [21].

Mice infected intravenously with amastigotes of *Leishmania donovani* or *L. infantum* have provided multiple insights into the fine detail of granuloma formation [23], as described in the following sections. Nevertheless, it is worth noting here that mice represent something of a hybrid when it comes to modeling the response observed in humans. Whereas the granulomatous response in the hepatic microenvironment appears to follow many of the characteristics observed in subclinical human and canine infection and in the ultimately self-resolving infection in primates, the spleen fails to make a robust granulomatous response and shows hallmarks of progressive human, primate, and canine disease. This expression of organ-specific immunity, within the same genetically and microbiota-defined host, provides a clear example of the compartmentalization of immune responses based on tissue-specific environmental factors [12, 24].

Post kala-azar dermal leishmaniasis is an important but relatively understudied sequelae of visceral leishmaniasis [25–28]. In Sudan, where post-kala-azar dermal leishmaniasis (PKDL) is commonly self-limiting, granulomas were reported in ∼20 % of patients, though other signs of chronic inflammation, e.g., the presence of giant cells, were more frequently observed [29, 30]. In India, where PKDL is less frequent but more chronic in nature, granuloma formation was notably absent, suggestive of a causative relationship between granuloma formation and host protection [31].

## Initiation of the hepatic granulomatous response in mice

Experimental murine models of VL have played an important role in understanding the development of granulomatous inflammation in the hepatic microenvironment. It should be emphasized at the outset, however, that this represents very much a model system and that many aspects of the biology of natural infection are omitted from the model. Natural sandfly-mediated transmission of small numbers of *Leishmania* metacyclic promastigotes into the dermis is replaced by the intravenous administration of large (often very large!) numbers of culture-derived promastigotes (most common when using *Leishmania chagasi*/*infantum*) or amastigotes (commonly used for *L. donovani*). Although new imaging approaches have highlighted the novel route of *Plasmodium* transmission to the lymphatics during mosquito blood feeding [32], our current knowledge of sandfly transmission of *Leishmania* suggests that parasites are deposited in the dermis, where they may be engulfed by neutrophils, dermal macrophages, or dermal DCs [3]. Sandfly salivary components also have a direct capacity to modulate local and perhaps systemic immune responses, and this may alter the hepatic microenvironment. Whether visceralisation of *Leishmania* to the liver and elsewhere is accomplished by free parasites (unlikely) or by migratory infected myeloid cells (more likely) has yet to be determined experimentally. In part, this technical challenge is one of numbers—tracing a few initiating foci of infected Kupffer cells within the myriad sinusoidal network of the liver is a major challenge.

**Fig. 1:**
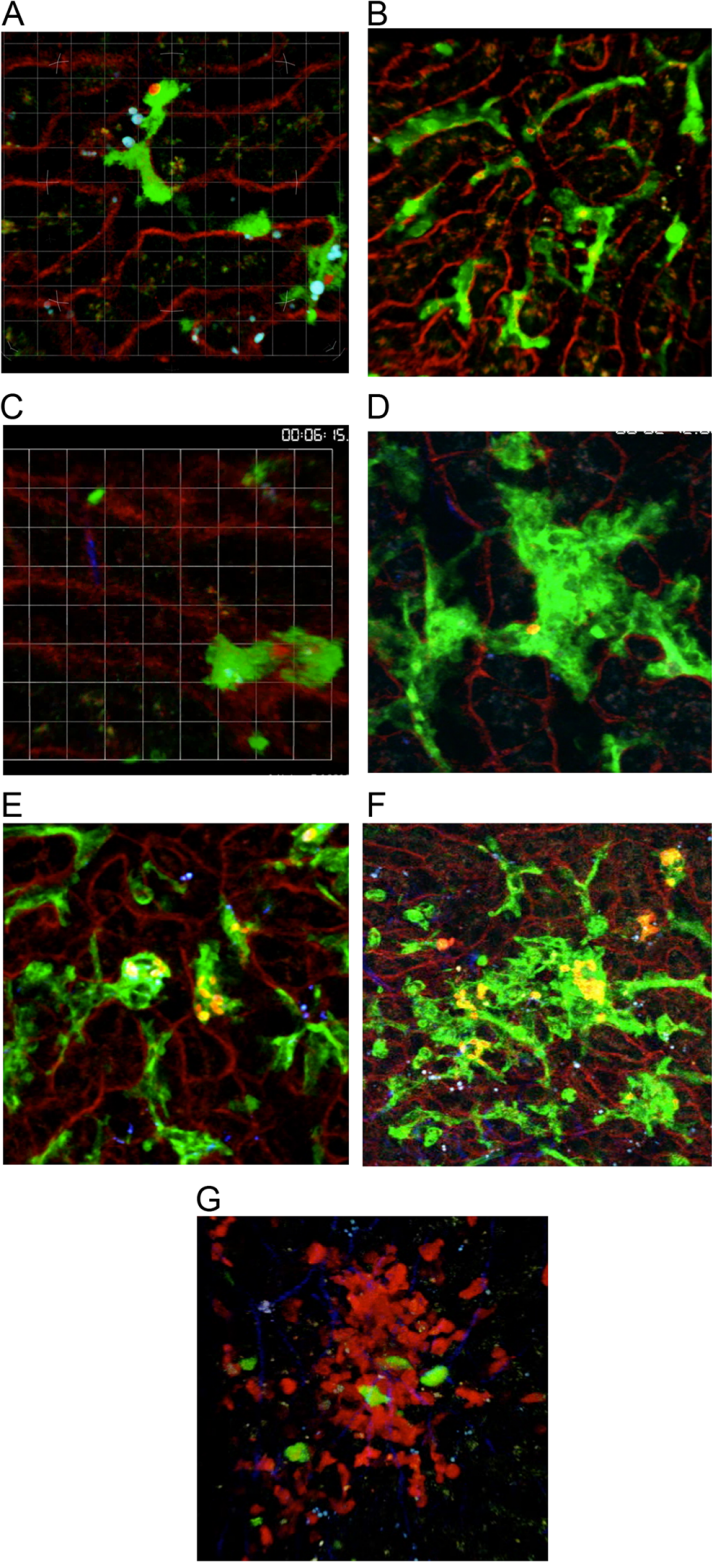
Intravital imaging of granuloma formation in experimental *L. donovani* infection. **a**–**g** Intravital analysis of KC (*green*) in liver sinusoids (*red*) in (mT/mG × LysMcre)_F1_ mice; **a** immediately following intravenous injection of tdTomato *L. donovani* amastigotes, **b** 2 h post-injection of tdTomato *L. donovani* amastigotes, **c** immediately following intravenous injection of tdTomato *L. donovani* metacyclic promastigotes, **d**–**f** 6 days post-injection of tdTomato *L. donovani* amastigotes. **g** CFSE-labeled memory-like OT-I T cells (*green*) 5–14 h post-transfer into d14-21-infected hCD2.RFP mice (red T cells) infected with OVA-transgenic *L. donovani*. All images represent snapshots of the extended focus view of time-lapse imaging sequences. Associated videos are provided as Supplementary Movies 1–7. For further experimental details, see references [36, 37].

Hence, what we know about the initiation of granulomatous inflammation in the liver begins from the somewhat artificial starting point of an interaction between free amastigotes and liver resident cells lining the sinusoidal spaces of the healthy non-inflamed liver. Under these infection conditions, the Kupffer cell is the primary target of *L. donovani* amastigotes. Early immunohistochemical analysis identified Kupffer cells on the basis of their characteristic morphology and anatomical position within the sinusoids [33]. Later, using real-time intravital 2-photon imaging, the uptake of amastigotes in situ was visualized for the first time. Kupffer cells are the resident tissue macrophages of the liver, originating from a wave of primitive yolk sac hematopoiesis that starts around embryonic day E7.5 and involves a *myb*-independent precursor [34, 35]. Using a LysMcre reporter mouse crossed to the dual-color reporter mouse line mT/mG, Beattie et al. demonstrated that Kupffer cells are stationary within the sinusoids and display elegant membrane activity, with continuously extending and retracting lamellipodia activity [36, 37]. Following contact with injected amastigotes, uptake is rapid and completed within minutes, with no obvious change in lamellipodia activity either at the contact site or distal to it (Fig. 1a; Supplementary Movie 1 and [36]. Phagocytosed amastigotes were observed to reside within intracellular vacuoles from the earliest timepoint that can be studied (Fig. 1b and Supplementary Movie 2; Beattie, unpublished).

Uptake of *Leishmania* by macrophages has been shown to be dependent on both host cell type and parasite species and involve distinct mechanisms in vitro [38, 39]. The intravital imaging data described above demonstrate that within the liver microenvironment, phagocytosis of amastigotes by Kupffer cells is a relatively passive process, displaying none of the indicators of induced phagocytosis or macropinocytosis. A currently unanswered, yet tractable question that follows from these imaging studies is the nature of the ligand responsible for amastigotes recognition and uptake. In vitro studies have identified a range of potential receptor ligand pairs involved in *Leishmania*–macrophage interactions and their relevance in vivo deserves to be explored more fully [38]. A recent study identified divergent roles for the C-type lectins SIGNR3, dectin1, and mannose receptor in determining the fate of *L. infantum*. Whereas SIGNR3 favored parasite survival through selective modulation of LTB4-IL-1β, both dectin1 and MR were coupled to signaling pathways associated with ROS production [40]. Although neutrophils were also observable patrolling the sinusoidal spaces in these imaging studies, they appeared to make little contact with *L. donovani* amastigotes and only rarely was phagocytosis directly observed. This is in contrast to the rapid engulfment of intravenously inoculated promastigotes of *L. donovani* by neutrophils (Fig. 1c and Supplementary Movie 3). The lack of immediate neutrophil swarming into the liver postinfection (a characteristic of promastigote inoculation; Beattie, unpublished) also confirmed the relative lack of inflammatory stimuli triggered by amastigotes under these conditions. Neutrophil indifference to amastigotes compared to promastigotes has also been reported for other *Leishmania* spp. in vitro [41].

Intravital imaging studies of BCG-infected mice showed a remarkably similar picture for the uptake of this mycobacterium; BCG was internalized by KCs within a minute or so of intravenous injection and became associated with intracellular compartments within 5 min of contact. Neutrophils, when observed patrolling the sinusoidal space, did not appear to engulf or contact BCG organisms in any appreciable manner, and this apparent indifference was also apparent even after the neutrophil influx characteristic of the early stages of BCG-induced inflammation [42].

## Chemokines and cytokines regulating early mononuclear cell recruitment

Mononuclear cell recruitment into the nascent granuloma can occur by local repositioning of resident Kupffer cells or through the initiation of inflammation. Early during the process of granuloma formation, Kupffer cells may fuse, resulting in multinucleation [23] (Fig. 1d and Supplementary Movie 4). This process involves both infected and uninfected Kupffer cells, implying cues for local detachment and migration along the sinusoidal endothelium. Such Kupffer cell motility was also observed using BCG infection, and in both model systems, areas of Kupffer cell-rich granulomatous inflammation become interspersed with areas of the sinusoidal network devoid in their resident macrophages [37, 42]. Notably, the fate of Kupffer cells and their propensity to either form multinucleate cells or aggregates appears independent of parasite load, with some heavily infected Kupffer cells remaining as singletons in the highly inflamed liver environment for protracted periods of time (Fig. 1e and f and Supplementary Movies 5 and 6). This evident heterogeneity underlying granuloma formation provides a context for the development of computational simulations that aim to provide a mechanistic understanding of this observation (see below). Comparison with the behavior of Kupffer cells in BCG and *L. donovani* granulomas also serves to emphasize the relatively static nature of these cells/aggregates once within the core of the granuloma [37, 42], and although it is not possible to formally rule out, under the imaging conditions used, that some singleton-infected Kupffer cells arise from migration of Kupffer cells out of the granuloma environment, as noted in the granulomas formed in zebrafish infected with *Mycobacterium marinarum* [43], there is no current evidence to support this from studies in the immunologically intact mouse.

As infection proceeds past the first few hours, inflammatory cells begin to be recruited to the infected liver, and more specifically to infected Kupffer cells. Conventional approaches to examining the factors responsible for regulating tissue recruitment of inflammatory cells involves measurement of total tissue-derived chemokines/cytokines. We and others have used this approach to show early chemokine production, notably CCL2, CCL3, and CXCL10 [44–48]. In some instances, the functional importance of these factors has been validated by the use of gene-targeted mice in which there has been global loss of production of the factor under study. In common with similar studies using BCG and MTB, these experimental approaches provide valuable information about the cellular environment underlying the inflammatory event but fail to provide information regarding the cell-specific nature of these responses. Chemokines in particular are difficult to study by conventional immunohistochemical techniques, limiting the spatial information available. In some cases, ex vivo analysis of chemokine production has been performed following flow cytometry, but this has been on a rather piece-meal gene by gene basis. The importance of spatial information is exemplified by our analysis of CXCL10 regulation. Messenger RNA (mRNA) accumulation for *Ccl2*, *Ccl3*, and *Cxcl10* was coordinately increased at 2 h postinfection in both wild-type mice and immunodeficient SCID or *Rag1*^−/−^ mice [44, 48], and *Ccl2* and *Ccl3* mRNA returned to baseline levels by 24 h in both strains of mice [44]. In contrast, wild-type but not immunodeficient mice exhibited sustained accumulation of *Cxcl10* mRNA, indicative of the IFNγ-regulated nature of this chemokine. Surprisingly, however, given that IFNγ-producing NK cells, non-invariant NKT, and invariant NKT cells could all be detected in the liver at this time, studies in Jα281^−/−^ mice indicated that only invariant NKT cells produced IFNγ that was functionally relevant in this context [48].

In order to determine the global Kupffer cell response to infection, we developed an ex vivo transcriptomic profiling approach, wherein infection with TdTomato-expressing transgenic parasites allowed us to rapidly sort both infected and uninfected Kupffer cells from the liver of the same infected mouse. By comparison to Kupffer cells isolated from infected mice, this provided a unique opportunity to not only identify transcripts associated with the presence of intracellular amastigotes, but also to reveal how Kupffer cell activation proceeds in trans in response to inflammation [36]. This technique identified transcriptional signatures common to infected and “inflamed” Kupffer cells (largely mapping to GO terms associated with host defense and inflammatory response and validating many of the chemokine pathways identified earlier) and also signatures unique for infected cells (often associated with the regulation of macrophage gene expression and protein production). Surprisingly, we identified 241 transcripts uniquely regulated in the “inflamed” but not infected population of Kupffer cells. One explanation for this finding was that parasitism was favored by maintaining these genes at homeostatic levels even in the face of inflammation; hence, these genes might regulate a presumptive host defense pathway the parasite is avoiding. To test this hypothesis, we used pharmacologic intervention to perturb one of these regulatory gene networks, involved in lipid metabolism, and demonstrated that indeed this led to more effective Kupffer cell control of amastigote replication [36].

Other studies have examined the impact of gene KO on early myeloid cell recruitment. *Ccr5*, *Ccl3*, and *Ccr2* KO mice all have KC aggregates of reduced size at 4 days p.i., with impaired granuloma organization measurable at 4 weeks [49]; similar defects were noted in a model of PPD-coated latex bead-induced lung granulomas in *Ccr2* KO mice [50] and also in *Ccr1* KO mice in the *Schistosoma* egg granuloma model [51].

IL-33 is a Th2-associated cytokine that belongs to the alarmin family and that signals via the ST2 (Il1rl1)/IL-1 receptor accessory protein complex. IL-33 is produced by innate lymphoid cells and also by many non-hematopoietic cells. CCL2 and CXCL2 and their receptors (CCR2 and CXCR2) are increased in expression in hepatic tissues in infected *Il1rl1* KO BALB/c mice coincident with enhanced Th1 responses and granuloma development and monocyte and neutrophil recruitment. These data suggest that signaling through ST2/Il1rl1 is a negative regulator of granuloma formation [52].

*Cxcr3* KO mice, defective in responsiveness to CXCL9 and CXCL10, have delayed granulomatous responses but show otherwise effective antimicrobial function [53]; A similarly delayed response to aerosolized MTB infection was noted in *Cxcr3* KO mice, suggesting that neutrophils were important for regulating early inflammation, though ultimately clearance of infection was obtained in these mice [54]; hence, *Cxcr3* was not essential for immunity to *L. donovani* but allowed optimal recruitment of leucocytes to the granuloma [53].

Jα281 KO mice defective in invariant NKT cells have delayed granuloma formation, and 90 % of all chemokine, cytokine, and associated receptor genes upregulated in the livers of wild-type (WT) *L. donovani*-infected mice were found to be underexpressed in similarly infected Jα18 KO mice, underscoring the global deficiency in early stages of granuloma formation in these mice [55].

The precise upstream signaling that drives these inflammatory chemokine responses in infected and inflamed Kupffer cells is poorly characterized. *Tlr2* KO mice infected with *Leishmania amazonensis* have eosinophilic granulomas in the early stages of infection compared to the mononuclear-rich responses of WT mice, suggesting that this TLR can modulate the balance of myeloid cell recruitment [56]. In *L. donovani* infection, TLR2 appears to negatively regulate granuloma formation, with more rapid granuloma maturation occurring in *Tlr2* KO mice; in contrast, TLR4 signaling appears to dampen the development of this response, and heavily parasitized granulomas persist. Importantly, the mechanisms of granuloma involution/resolution were not dysregulated in the absence of *Tlr2*, and such mice did not develop fulminating granulomatous pathology [57].

TLR9 is a PRR involved in the early recognition of *Leishmania* under some circumstances [58]. TLR9 expression has been demonstrated in granulomas from *L. braziliensis* patients but the evolution of this response was not studied and may relate to TLR9 regulation on macrophages after granuloma formation [59]. TLR9 has also been implicated in the initial response to *L. infantum* promastigotes in mice, acting as part of a regulatory network that controls NK cell IFNγ production. The impact of *Tlr9* deficiency on granuloma formation was not, however, reported [58].

IRF5 can be activated downstream of TLR9/Myd88 and TLR7/Myd88 signaling. *Irf5* KO mice infected with *L. donovani* have delayed Th1 responses, higher liver parasite burdens, and smaller Th2-biased granulomas with greater evidence of neutrophil involvement and concomitant accumulation of *Cxcl1* mRNA in the granuloma [60]. A similar phenotype was also noted in *Tlr7* KO mice. Although TLR7 operates via IRF7 as well as IRF5, no effect on splenic or Th1 responses (measured as CD4^+^ T cell-derived IFNγ in the liver or spleen) was noted as a consequence of *Irf7* deficiency in this study. In contrast, another study using *Irf7* KO mice demonstrated a reduction in granuloma formation, Th1 response, and local NO response, along with an increase in the frequency of CD4^+^ T cells committed to producing IL-10 [61]. Exemplifying the divergent mechanisms of tissue and cellular control of *Leishmania*, *Irf7*-deficient Kupffer cells were shown to have an intact innate response to amastigotes of *L. donovani*, compared to the defective response seen in splenic marginal zone macrophages [61].

Given its role in regulating the Th1 response, the finding that *Tbet*-deficient mice are highly susceptible to *L. donovani* infection was not unexpected. Although granuloma formation proceeds in these mice, albeit in a restricted manner, most measures of Th1 immunity are significantly compromised. In contrast, and unexpectedly, *Stat1*-deficient mice are highly resistant to *L. donovani* infection, with minimal or absent granuloma formation in these mice [62]. Of note, granuloma formation was equivalent in *Rag2* KO mice that received wild type or *Stat1*-deficient T cells by adoptive transfer, demonstrating that induction of granulomatous pathology was regulated through STAT1-dependent pathway(s) operating in non-lymphoid cells [62].

Although limited in the granulomas of wild type *L. donovani*-infected mice, there is increasing evidence that neutrophils regulate granuloma formation in experimental VL. Delayed granuloma formation was observed in mice depleted of neutrophils using mAb NIMP-R14, but this was far less of an effect than that observed on parasite load in the spleen and BM [63]. In B cell-deficient μMT mice, neutrophilic infiltration was exaggerated, leading to widespread hepatic necrosis, rarely seen in wild-type mice infected with *L. donovani*. In the context of B cell deficiency, neutrophil-mediated pathology could be ameliorated by passive transfer of Ig [64].

## The maturing granuloma: a focus for cell-mediated immunity

Analysis of the cytokine response in the maturing hepatic granuloma has been the focus of intense study for over three decades [65], and extensively reviewed on multiple occasions, often contrasting the host protective granulomatous response with the non-protective and non-granulomatous response seen in the spleen [12, 23, 24, 66, 67]. The reader is referred to these articles for a current overview. In summary, granulomatous inflammation appears the preferred route for focusing cytokine secretion onto infected macrophages, with the aim of stimulating the production of toxic anti-leishmanial species including nitric oxide and superoxide. Although Th1-like responses dominate (with IFNγ being produced by multiple cell types) and alternatively activated macrophages are sparse in the *L. donovani* granuloma, the Th2-associated cytokines IL-4 and IL-13 are nevertheless required for optimal granuloma formation [68, 69]. As Kupffer cells contain the vast bulk of amastigotes in the murine hepatic granuloma [37], it is likely that this macrophage population is responsible for parasite elimination. However, it remains possible that Kupffer cell lysis occurs and that leishmanicidal activity is delivered by granuloma-infiltrating monocytes or neutrophils that rapidly phagocytose the released amastigotes.

It is worth bearing in mind, however, some of the experimental limitations of many of these studies, when it comes to precisely assigning the role of cytokines and/or cells within the microenvironment of the granuloma per se. For example, technical challenges associated with the small size and limited structural integrity of the *L. donovani* granuloma makes isolation difficult and often whole liver cell populations are used for analysis as a surrogate. Alternatively, the experimental intervention may affect immune regulation within the granuloma, but it also likely has more global effects (e.g. consider the mouse treated with mAb to neutralize a given cytokine), that indirectly affect granuloma form or function. Finally, while granuloma heterogeneity (cellularity, location, or apparent microbicidal activity) is clearly evident even by simple histological analysis, aggregation of data from this heterogeneous population is the current norm. Unlike studies on TB, where ex vivo access to and/or imaging of individual granulomas in situ is possible [70–72], our ability to interrogate the individual granuloma in leishmaniasis patients and animal models remains limited at present.

Attempts to isolate the hepatic environment from other systemic sites responding to their own “local” *Leishmania* infection have provided some insights into the independence of hepatic immunity, but largely not addressed the challenge of defining responses within the granuloma microenvironment per se. For example, in *aly*/*aly* mice that lack LN, Peyer’s patches, and have disrupted splenic architecture, granulomas are generated in the liver [73]. This is in keeping with recent data showing that splenectomized LT-deficient mice (also lacking LNs, hence devoid of conventional secondary lymphoid tissue) have the capacity to mount immune responses against this infection including normal granuloma formation [74]. In the case of *aly*/*aly* mice, parasites persist within granulomas accompanied by an influx of Foxp3^+^ Tregs that is not seen in granulomas of WT mice [73].

Intravital microscopy provides the opportunity to study individual granulomas in situ in live animals, but imposes its own constraints. Imaging windows are short, usually a few hours at most, and the procedures used are terminal, limiting the observation of granulomas over realistic time periods to observe their maturation. Observation is generally limited to one or a small number of cell populations, and the functional consequences of those interactions, be that intracellular signaling events or cytokine production, are only recently becoming amenable to imaging [75]. Nevertheless, the study of CD4^+^ T cell [42, 76, 77] and CD8^+^ T cell behavior within the BCG and *L. donovani* [37, 78] granuloma has provided valuable novel insights into local effector T cell activity (Fig. 1g and Supplementary Movie 7). In spite of these studies focusing on different T cell subsets, commonalities emerge: (i) both antigen-specific and non-specific T cells are recruited into the granuloma—a not unsurprising finding given the inflammatory nature of this environment, but one that serves as a reminder that enumeration of all T cells in the granuloma is not a likely measure of effector function; (ii) T cells move freely into and out of granulomas, with no evident walling-off of the granuloma from its external environment; (iii) within granulomas T cell velocity was similar to that seen in organized lymphoid tissues, but other measures of T cell migration indicated some local constraints on free migration within the confines of a granuloma; (iv) long-term contacts between T cells and APC, indicative of cognate interactions and measurable by a dwell time for granuloma exit, was observed for a minority of antigen-specific T cells, suggesting local presentation of cognate antigen was limiting. This conclusion is supported in the case of CD4^+^ T cells by demonstrating arrest of a much greater frequency of intra-granuloma T cells after exogenous administration of peptide [76]. Within the *L. donovani* granuloma, expression of MHCI-peptide complexes was an almost exclusive property of infected Kupffer cells and such cells formed the focus of attention for CD8^+^ T cells [37]; and (v) constraints on APC efficiency within the granuloma may reflect limited MHC-peptide complex expression coupled to local pathogen load [76, 77] or altered expression of checkpoint inhibitors [79].

These studies provide a basis for future examination of other aspects of granuloma biology that may be pertinent to understanding disease chronicity and or targeted therapeutic intervention. For example, whether T cell behavior remains similar across the spectrum of granuloma heterogeneity is unknown, and how changing features of the non-lymphoid compartment of the granuloma including progressive fibrosis, may affect T cell–APC interactions would appear to be an essential route of future investigation. Likewise, the role of checkpoint inhibitors as host-directed therapy has a proven experimental base (see below) but whether this occurs through direct manipulation of T cell biology in the granuloma remains to be established. As pointed out by Germain and colleagues [76], manipulation of local APC function may prove more profitable than or be an effective adjunct to enhancing effector T cell frequency, and in situ analysis of the type described above may provide the most useful means to monitor the success of such interventions.

## Computational simulation of granuloma development and function

To overcome some of these experimental limitations and to gain further insight into granuloma heterogeneity, dynamics, and function, we and others have also adopted computational approaches. In TB, these models have focused largely on understanding macrophage polarization and the role of TNF [80–82].

Our approach in modeling the *L. donovani* granuloma has been twofold—first, the generation of agent-based models of early granuloma formation and, second, the use of Petri net modeling of granuloma heterogeneity and within granuloma immune interactions. The former have been discussed elsewhere [83] and will not be considered further here. The Petri net model that we have used to evaluate the development of granulomas and the emergent immune regulation that influences parasite load is not spatially explicit and assumes no structural organization within the granuloma per se. It has, however, provided invaluable insights into potential sources of granuloma heterogeneity and allowed us to postulate the presence of a small subset of granulomas that have altered immune regulatory function that leads to parasite persistence [84]. Such granulomas may well be a source of recrudescence upon immune suppression, a common feature of experimental models of VL and also seen in human disease, and the generation of new hypotheses amenable to experimental validation is arguably one of the central roles that modeling can play in understanding complex, dynamic, and long-lived disease processes.

## The granuloma as a tertiary lymphoid organ

Tertiary lymphoid organs are commonplace in conditions of chronic inflammation, representing accumulations of lymphoid cells and stromal cells that recapitulate aspects of lymphoid tissue architecture [85]. In autoimmune diseases, the production of tertiary lymphoid organs (TLOs) is increasingly recognized as a poor prognostic indicator, serving to perpetuate the immune response. Lung TLO’s have also been reported in mice infected via aerosol with *Mycobacterium tuberculosis* H37RV and in human tuberculosis, associated with granulomas [86–88]. Central to the formation of TLOs is the lymphotoxin-dependent appearance of stromal cells producing constitutive chemokines associated with lymphocyte aggregation. Notably, most TLOs express CXCL13, the chemokine produced by follicular dendritic cells and responsible for B cell aggregation in germinal centers [89]. Hence, curtailment of the production and maintenance of TLOs is viewed as a potential target for stromal cell-targeted host-directed therapy. Although B cells are found within the granulomas of *L. donovani*-infected mice, and these make sustained interactions with granuloma T cells that are suggestive of cognate interactions [90], we have not found evidence for TLO-like organization either within the granuloma itself or within the hepatic microenvironment during *L. donovani* infection. CXCL13 expression was not detectable nor were cells expressing the FDC marker FDCM1. The reasons for the lack of TLO formation during *L. donovani* infection are currently unknown. One possibility is that the relative paucity of strong PAMPs in *Leishmania* compared to MTB limits the extent of underlying innate immune responses that drive TLO formation. On the other hand, the relative ease with which *Leishmania* succumb to macrophage leishmanicidal activity may allow premature truncation of the infection before TLOs can fully develop. Alternatively, *Leishmania* infections may not provide appropriate signals for the recruitment and activation of lymphoid tissue inducer (LTi) cells. LTi cells are a necessary precursor to TLO formation. In support of this latter possibility, whereas restoration of lymphoid tissue architecture following MCMV infection is dependent upon LTi function [91], such cells do not appear to play a role in restoration of lymphoid tissue architecture in *L. donovani*-infected mice treated with sunitinib maleate, a receptor tyrosine kinase inhibitor that restores stromal cell structure and function [92].

## Host-directed therapies targeting granulomatous inflammation

The therapeutic benefit of modulating key pathways in immune regulation on the outcome of *L. donovani* infection provided some of the earliest examples of the use of checkpoint inhibitors, now an increasingly popular clinical intervention strategy for cancer and autoimmunity [93, 94], as tools to manipulate immunity to pathogen infection. Following observations that the expression of costimulatory molecules of the B7 family were reduced in expression on infected Kupffer cells at the core of the granuloma [79] and on *L. donovani*-infected macrophages in vitro [95], mAb targeting costimulatory pathways were examined for their ability to affect granuloma formation and host protective immunity. Administration of a blocking antibody to B7-2 (CD86) had no impact on early immune or inflammatory responses, whereas later administration led to enhanced Th1 responses and granuloma formation. These data suggested that late CD86-CTLA4 interactions might limit the expression of maximum immunity to *L. donovani* [96]. Confirming this prediction, administration of a mAb (4F10) targeting CTLA-4 was shown to significantly accelerate granuloma formation and lead to significant reductions in tissue parasite burden. Notably, anti-CTLA4 could be used therapeutically in mice with established infection, either alone [97] or in a synergistic fashion with conventional chemotherapy [98, 99]. Other checkpoint regulators including OX40L-Fc [98], anti-CD40 [99], and PDL-1 [100] have proven equally effective at enhancing granuloma formation and protective immunity in chronically infected mice, in the absence of any overtly tissue-damaging pathology. To date, no clinical trials of such checkpoint inhibitors have been performed in the context of leishmaniasis, although evidence for defects associated with dysregulated checkpoint control, e.g., CD8^+^ T cell exhaustion, have been obtained in the clinic [101]. Attempts to validate the use of checkpoint-regulating antibodies with in vitro cultures of infected macrophages and T cells have had mixed results, with the PD-1 pathway blockade inducing heightened macrophage activation and parasite killing in cells obtained from active cases of canine VL [102] but not human VL [101].

The success of checkpoint blockade in models of *L. donovani* infection is not mirrored in models of mycobacterial infection. For example, whereas CTLA4 blockade significantly increased multiple correlates of immunity in mice infected with BCG, there was no impact on mycobacterial load in the spleen, liver, or lung and no changes to lung histopathology and granuloma formation [103]. Importantly, removal of regulatory control over T cell activation during mycobacterial infection may lead to the development of overt pathology, as in the case of manipulation of the PD-1 pathway [104]. It is likely that differences in outcome of checkpoint regulation related to pathology vs. protection reflect the relative ease with which *Leishmania* succumbs to the antimicrobial defenses compared to mycobacteria and to differences in the inherent immunogenicity of these organisms, with *Leishmania* being a renowned “silent invader” [3]. These characteristics may suggest a brighter future for checkpoint inhibitors as therapeutic agents in human leishmaniasis compared to mycobacterial disease.

## Electronic supplementary material

Movie 1 (MPG 4.38 mb)

Movie 2 (MP4 4.26 mb)

Movie 3 (MP4 6.88 mb)

Movie 4 (MP4 13.9 mb)

Movie 5 (MP4 8.10 mb)

Movie 6 (MPG 8.47 mb)

Movie 7 (MP4 7.20 mb)
